# High productivity of tree species planted outside their current geographic range indicates large regions of unrealized niche space

**DOI:** 10.3389/fpls.2025.1650428

**Published:** 2025-08-28

**Authors:** Hardy Griesbauer, Gregory A. O’Neill, William H. MacKenzie

**Affiliations:** ^1^ British Columbia Ministry of Forests, Omineca Region, Prince George, BC, Canada; ^2^ British Columbia Ministry of Forests, Kalamalka Forestry Centre, Vernon, BC, Canada; ^3^ British Columbia Ministry of Forests, Smithers, BC, Canada

**Keywords:** assisted range expansion, forest productivity, realized niche, fundamental niche, assisted migration, silviculture, climate change, species distribution modeling

## Abstract

**Introduction:**

Reforestation efforts that utilize assisted range expansion in response to climate change require an enhanced understanding of tree species’ fundamental niches as well as potential interactions with local species when planted in novel environments.

**Methods:**

Using height-age modeling and dendrochronological approaches, we analyzed height and radial growth data from 25 disparate experimental plantings of three temperate zone conifer species (Douglas-fir, western larch and ponderosa pine) in environments north of, and colder than, their current geographic range in British Columbia, Canada, to explore how these types of trials can provide important insights into tree species’ fundamental niches in regions that lack comprehensive, replicated experiments.

**Results and discussion:**

Height growth of the three species exceeded or equaled that of populations growing within their realized niche, suggesting that from a growth perspective, the fundamental niches of these species have historically included environments colder and further north than their current ranges. The productivity of Douglas-fir and western larch also exceeded that of a local tree species, lodgepole pine, across a range of sites and climates within the study region, indicating that these species may be successful competitors with other species outside their range. Across all species and locations, environmental constraints on tree productivity appear to be more consistently related to available soil moisture than temperature. This study suggests that temperate conifers may have unrealized niche space far outside their current geographic range, and their potential for range expansion may be larger than suggested by species distribution models.

## 1 Introduction

Climate is the primary factor controlling the broad-scale distribution of plants (Woodward, 1987). However, habitat fragmentation, long periods required for trees to reach reproductive maturity, short seed dispersal distances, and inter-specific competition can create barriers that constrain a tree species’ realized niche (i.e., its current geographic distribution) to an area smaller than its fundamental niche (i.e., where the species could grow) (Hargreaves et al., 2014; Laughlin and McGill, 2024). The rate of climate change is expected to outpace the rate at which conifer populations can migrate (Ash et al., 2017), resulting in a mismatch between the climate where a population (or species) is found and its optimal climate (O’Neill et al., 2008). Climate mismatch can predispose forests to widespread maladaptation (Aitken et al., 2008; Rehfeldt et al., 2014); symptoms of climate change-related forest maladaptation may include reduced growth, poor reproduction, reduced resistance to diseases and insects, and increased mortality (Boisvenue and Running, 2006; Allen et al., 2010).

Movement of tree populations and species during reforestation to their cooler historic climates within, and outside, their current geographic range (i.e., assisted population migration, and assisted range expansion, respectively) have been widely discussed as strategies with the potential to mitigate some of the impacts of climate change-related forest maladaptation (Williams and Dumroese, 2013). These strategies may help ensure newly established forests continue to provide essential ecosystem goods and services, including carbon sequestration, nutrient cycling, timber, and wildlife habitat (Wang et al., 2006; Williams and Dumroese, 2013). The success of these strategies will require an understanding of a tree species’ realized and fundamental niches (Hargreaves et al., 2014; Laughlin and McGill, 2024), recognizing that quantifying the true fundamental niche for most tree species is exceedingly difficult. While numerous tools (MacKenzie and Mahony, 2021; St. Clair et al., 2022) and policies (O’Neill et al., 2017; van Kerkhof et al., 2022) have emerged to facilitate assisted population migration, there exists a paucity of data and approaches to adequately quantify a tree species’ fundamental niche, and therefore assess its feasibility for assisted range expansion. Several species distribution modeling efforts suggest species will perform well when planted in locations slightly outside their current range (Gray and Hamann, 2013; MacKenzie and Mahony, 2021); however, these models are largely limited to data collected from within the species’ realized niche and therefore may underestimate the fundamental niche (and consequently the potential for assisted range expansion) of these species. Further, most species distribution models use presence/absence as the response variable, which provides a limited understanding of how fitness varies outside a species’ current range (Gray and Hamann, 2013; Hargreaves et al., 2014; Zhao et al., 2024). The use of continuous variables such as growth *en lieu* of presence/absence data may improve species distribution modeling and provide an enhanced understanding of how fitness varies across a species’ fundamental niche (Zhao et al., 2024).

Species’ fundamental niches are best informed by empirical evidence of fitness in trials which quantify species performance across a range of climates and sites; however, few comprehensive species trials exist in North America. Provenance trials that quantify growth or survival among multiple populations of a tree species across a range of climates would also provide robust empirical data to quantify the fundamental niche (and hence the potential for assisted range expansion) of a species (e.g., Wang et al., 2006). However, few provenance trials test species outside their current geographic range, and even fewer provenance trials test species outside of their current climate range, notable exceptions being the Assisted Migration Adaptation Trial in western North America (Marris, 2009) and the Reinforce trial in western Europe (http://reinfforce.iefc.net). Further, the few trials that have established species outside their current range are primarily designed to address questions related to moving a tree species or population to a new location without considering site edaphic conditions which can strongly influence the viability of species (MacKenzie and Mahony, 2021). Support for assisted range expansion would be further advanced with the establishment of a network of new trials that test the species across a range of edaphic conditions; but such trials would not produce actionable results for at least a decade.

With a paucity of provenance trials to inform the potential for assisted range expansion of tree species, the best source of empirical evidence may be found in less formal occurrences of tree species planted outside their current geographic range (Booth, 2024; Laughlin and McGill, 2024). In British Columbia (BC), Canada, individual foresters, forest companies and government agencies have established informal species trials to assess tree growth and survival in new locations. Many of these trials are unreplicated, lack a rigorous experimental design, and use a single population of each species, which limits their use in identifying the best seed source for a given climate. However, these trials often include local species for comparison purposes, and in some cases, these trials are many decades old, thus offering an opportunity to compare long-term growth between species (LePage and McCulloch, 2011).

Lodgepole pine (*Pinus contorta* Douglas ex Loudon) is used widely in reforestation in our study area in central BC, due to it being a native species in most of the area with broad ecological amplitude, desirable wood characteristics, and rapid juvenile growth. Studies predict that climate change will negatively impact the productivity of local populations of lodgepole pine in this region (Wang et al., 2006). Further, major biotic disturbances such as the mountain pine beetle (*Dendroctonus ponderosae* Hopkins) epidemic, which recently killed substantial areas of mature lodgepole pine stands in western North America (Burton, 2010), together with the potential for increasing frequency of lethal climatic disturbances, such as drought events (Zhao et al., 2020), warrant examination of additional species as candidate species diversification options (Millar et al., 2007). Consequently, the assisted range expansion of temperate zone tree species into the study area may help maintain the health and productivity of planted forests while providing options for increasing species diversity to help buffer potential effects of climate change (Mason et al., 2012).

Western larch (*Larix occidentalis* Nutt.) and ponderosa pine (*Pinus ponderosa* Lawson & C. Lawson) occur naturally only in southern BC (Klinka et al., 1999). The northern limit of the current geographic range of interior Douglas-fir (*Pseudotsuga menziesii* var. *glauca* (Mirb.) Franco) extends into the study area at low elevations (DeLong, 1996). These species are considered drought tolerant (Klinka et al., 1999) and bioclimate modeling suggests they will be well-adapted to projected future climates in parts of central BC and may be candidate species for assisted range expansion (Rehfeldt and Jaquish, 2010; Gray and Hamann, 2013; MacKenzie and Mahony, 2021) in the study area. Provincial planting standards were modified in BC in 2010 to allow western larch to be planted in portions of the west central region of the province, making it the first tree species in BC to be legally permitted for use outside its current geographic range (Nicholls, 2018). Modelled projections of suitable climate habitat for western larch and strong growth in long-term provenance trials (Rehfeldt and Jaquish, 2010) and unofficial trials (LePage and McCulloch, 2011) in this area supported this policy change.

In this study, we analyze growth data from 25 sites in central BC, Canada, where interior Douglas-fir (hereafter, Douglas-fir), western larch or ponderosa pine were established experimentally in locations outside and north of their current geographic range. This study seeks to address two main research objectives:

1. to examine variation in tree productivity across a range of environments located within and outside their current natural geographic range; and
2. to understand how measures of tree productivity can help elucidate the potential of temperate zone conifers planted outside their current geographic range to compete with local, naturally occurring tree species that are presumably well-adapted to local conditions.

This research also seeks to demonstrate the potential value of informal species trials in areas lacking extensive and replicated species or provenance tests to inform the suitability of assisted range expansion.

## 2 Methods

### 2.1 Field sampling

We identified 40 forest plantations in central British Columbia (BC), Canada, containing one or more of our three study species planted outside their current geographic range (OCGR). As the planting of tree species OCGR is rare in BC reforestation, we identified plantations for sampling using a combination of local knowledge, technical reports (LePage and McCulloch, 2011; Newsome et al., 2016) and the British Columbia provincial government’s RESULTS database ( *https://catalogue.data.gov.bc.ca/dataset/results-planting* *)*, which contains reforestation information (including tree species, seed source, planting date, and planting density) for publicly-managed forest plantations in the province. We selected 25 of the 40 candidate test sites for sampling based on accessibility, age (> 15 years), and trials with sufficient individuals to allow for quantitative evaluation of performance. Three of the test sites were established by BC government researchers as a trial examining the potential of tree species planted OCGR (Newsome et al., 2016), while the remainder were established as informal trials or demonstrations. Of the 25 test sites, 21 contained a single species planted OCGR and four contained two species planted OCGR. All sites but one were also planted with lodgepole pine as a comparison species. Field data were collected in autumn of 2020, 2021, and 2024. At each site, 4 to 13 circular plots with a 3.99 m radius (50 m²) were established in areas that were representative of the site and that included individuals of at least one test species and/or lodgepole pine.

Ecological information on soils, topography and vegetation communities was collected at each plot, following BC’s biogeoclimatic ecosystem classification system, to allow for identification of the site’s broad climatic regime (termed a ‘biogeoclimatic zone’) as well as the plot’s local ecological conditions (termed ‘site series’) (MacKinnon et al., 1992). Study sites were located in three biogeoclimatic zones: (i) Sub-Boreal Spruce, (ii) Interior-Cedar Hemlock, and (iii) Engelmann Spruce - Subalpine Fir. The actual soil moisture regime is an estimate of soil water balance throughout the year, calculated by estimating the ratio between actual and potential evapotranspiration (MacKinnon et al., 1992; DeLong et al., 2019), and is related to tree productivity (Wang and Klinka, 1996; Griesbauer et al., 2021). We identified actual soil moisture regime for each plot using modeled estimates for biogeoclimatic variants and site series in BC (https://catalogue.data.gov.bc.ca/dataset/forest-drought-risk-assessment-tool). The approach used to generate these estimates is described in DeLong et al. (2019), and the moisture regime classes used in this study are presented in **Appendix 1**. Plot actual soil moisture regime in this study ranged from moderately dry to fresh.

Within each plot, stem diameter at 1.3 m (i.e., diameter at breast height; DBH) was recorded on all live trees having a DBH equal to or exceeding 4 cm. Total tree height was recorded using a vertex hypsometer. Stem form traits, including crooks and forks, and forest health factors were also noted for each tree. Increment cores were taken at a height of 1.3 m on the tree bole from approximately 15 trees of the local species (lodgepole pine) and each of the non-local species (western larch, Douglas-fir, and ponderosa pine) at each site. Sampled trees were selected from among the dominant or co-dominant crown class and were free of obvious forest health factors or stem defects that might have affected growth. A single increment core, positioned perpendicular to the slope to minimize the presence of compression and tension wood on the tree-rings contained in the core, was taken from each tree.

### 2.2 Dendrochronological preparation

Increment cores were glued to wooden mounts and sanded with progressively finer sandpaper to expose the boundaries between annual growth rings. Cores were scanned with an optical scanner and ring widths were measured to 0.01 mm precision using CooRecorder software (v9.3.1, Larsson, 2014). Calendar years were assigned to each tree ring using visual crossdating techniques (Fritts, 2012), which were confirmed using statistical crossdating in the CDendro software (v9.3.1, Larsson, 2014).

Annual basal area increment was estimated by calculating distance from the pith to each ring using the *dplR* R package (v1.7.2, Bunn et al., 2022). Where cores missed the pith, the distance to the pith was estimated using the distance to pith estimation function in CooRecorder. For cores that did not contain the pith, the number of missing rings was also estimated by dividing the distance to the pith by the mean width of the three rings closest to the pith. This allowed for the tree age at core height to be estimated and allowed us to assign an age for each tree ring.

### 2.3 Site index estimates

To address the objectives of this study we analyzed long-term height growth. Because we sampled trees across various ages, we used height-age models to estimate tree total height at a common breast height age (hereafter, age) of 50. This is equivalent to the concept of site index, which is used to estimate the productivity of a tree species for a given site (Oliver and Larson, 1996). Site index estimates for trees growing within the species’ current geographic range (WCGR) were obtained from records for individual trees in the BC Site Index Estimates by Biogeoclimatic Ecosystem Classification Site Series (SIBEC) database (Mah and Nigh, 2003). The SIBEC program was initiated in 1994 to provide species-specific height growth estimates across a range of climatic regimes and site-level ecological conditions, including soil moisture regime (Mah and Nigh, 2003). Field data collection under SIBEC protocols consists of measuring an individual tree within a circular plot that is considered to best represent the species’ potential growth for a given site. Tree selection criteria include: (i) the largest diameter tree in a SIBEC plot is considered the best representative of growth potential; (ii) selected trees must occupy a dominant/co-dominant crown class (to reduce potential effects of inter-tree competition on height growth); and (iii) selected trees must be free of forest health factors or stem defects that could affect growth (Mah and Nigh, 2003). Site index estimates in the SIBEC database and for trees in the study area were derived from species-specific growth intercept models for trees with an age under 50 years and species-specific conventional site index models for trees with an age greater than 50 years (Mah and Nigh, 2003). Site index estimates for all trees were computed using version 4.4 of the BC Ministry of Forests’ Site Tools model ( *https://tinyurl.com/2bmyv7fa* *)*, set for the interior region of BC.

### 2.4 Climate data

Annual climate variables for the 25 selected test sites as well as records in the SIBEC database were obtained for the 1981–2010 climate normal period – the period that best represented the growth period of the majority of the plantations - by inputting site geographic coordinates and elevation into ClimateBC (v7.2, Wang et al., 2012). Two variables commonly used in forest productivity models were used to represent site climate: growing-degree days above 5°C (DD5) and climatic moisture deficit (CMD). See Wang et al. (2012) for details regarding these climate variables. While provenance can strongly influence productivity of planted stands (Leites et al., 2012; Sattler et al., 2024), a lack of provenance information in our data precluded genecological analyses.

### 2.5 Analyses

All statistical analyses were conducted using the R language for statistical computing (v4.1.3, R Core Team, 2022). Due to the nested structure of the data, we employed a linear mixed effects modeling approach to quantify species productivity, using the *lmerTest* R package (v3.1.3, Kuznetsova et al., 2017). Contrasts and estimated marginal means were computed using the *emmeans* R package (v1.7.3, Lenth, 2022). The proportion of variance explained by each model was calculated following Nakagawa and Schielzeth (2013), as implemented in the *MuMIn* R package (v1.46, Bartoń, 2023). Model residual plots were used to evaluate the model goodness of fit, as well as normality and homogeneity of variance among residuals. Significance is reported at *p*< 0.05.

#### 2.5.1 Analysis 1 – comparison of productivity inside and outside the species’ current geographic range

To compare species’ productivity between locations OCGR and WCGR, we developed a linear mixed effects model using each tree’s estimated site index as the dependent variable, and its location (OCGR or WCGR) as a fixed effect. For species OCGR (i.e., within the study area of central BC), we selected the largest-diameter individual per species in each plot that met SIBEC tree selection criteria (discussed previously). We controlled for the influence of environmental factors on site index by including site climate (DD5 and CMD) and plot-level actual soil moisture regime (ASMR) as additional fixed effects. In preliminary analyses, we fit the data with a model that included a random intercept varying among sites. However, examination of the residuals indicated poor overall fit, caused predominantly by the SIBEC data, which often contains only one site index estimate per site. A subsequent fixed effects model (without any random effect) was developed and found to have improved fit and residual diagnostics. This fixed effect model had the following form:


(1) $$SI_{ij}={\text{θ}}_{0\;}+{\text{θ}}_{1\;}Location_{i}+{\text{θ}}_{2\;}DD5_{i}+{\text{θ}}_{3\;}CMD_{i}+{\text{θ}}_{4\;}ASMR_{ij}+e_{ij}$$

where estimated site index of tree *j* in site *i* is the response variable and is denoted by *SI_ij_*. Model fixed effects beyond the intercept (θ_0_) are denoted by: (i) the tree’s location (WCGR vs OCGR) of site *i* (*Location_i_*); (ii) growing degree days above 5°C of site *i* (*DD5_i_*)*;* (iii) climatic moisture deficit of site *i* (*CMD_i_*); and (iv) actual soil moisture regime of tree *j* in site *i* (*ASMR_ij_*). In a preliminary analysis, we included interaction terms between *Location* and the three environmental covariates (i.e., *DD5, CMD*, and *ASMR*); these interactions were not included in the final model because the majority lacked statistical significance and graphical analysis indicated they were not necessary to explain variation in the response variable. The experimental error is denoted with *e_ij_.*

#### 2.5.2 Analysis 2 – comparison of productivity among species in the study area

The second analysis compared productivity of the three test species and the local control species (lodgepole pine) when growing within the study area. In this analysis, we developed separate models to analyze three measures of productivity: (i) long-term height growth, using site index estimates (described above); (ii) early tree growth, estimated as the number of years to reach breast height (YBH) after planting; and (iii) long-term radial growth, using basal area increment (BAI). Early tree growth is an important measure of a tree’s adaptation to site climate and ability to compete with co-occurring trees and vegetation during establishment. Radial growth is sensitive to climate (Fritts, 2012) and is an important component of estimating tree yield.

##### 2.5.2.1 Site index model

The process to derive site index estimates is discussed in the previous section, however, this analysis of site index differed from the first analysis in that we included site index estimates for all individuals (i.e., not just the largest-diameter individual) in a plot with a dominant/co-dominant crown class and with good form. Site index estimates for all species were pooled into a model with the following form:


(2) $$SI_{ijk}={\text{θ}}_{0\;}+{\text{θ}}_{1\;}Species_{k}+{\text{θ}}_{2\;}BGC\;Zone_{i}+{\text{θ}}_{3\;}ASMR_{ij}+u_{i}+v_{ij}+e_{ijk}$$

where *SI_ijk_* is the estimated site index of tree *k* in plot *j* of site *i*. Model fixed effects beyond the intercept (θ_0_) are denoted by: (i) tree species (*Species_k_*); (ii) the biogeoclimatic zone of the site (*BGC Zone_i_*); and (iii) actual soil moisture regime of the plot (*ASMR_ij_*). Preliminary analysis using site climate variables DD5 and CMD resulted in poor model performance; therefore, we used a broader climatic characterization (i.e., the site’s biogeoclimatic zone) to represent site climate. The model included two independent Gaussian random effects *u_i_* and *v_ij_* to account for the (pooled) variation among site and plots (nested within site). The residual error is denoted by *e_ijk_.*

##### 2.5.2.2 Years to breast height model

The YBH response variable was calculated by subtracting breast height age from the total age of the stand at the time of assessment. The total age of the stand was determined using the recorded planting date. We did not adjust the age of the stand for seedling age at the time of planting, as those data were not available for all planted stock. This model was specific to the study region, and data from all three test species and the control species were pooled into a single model with the following form:


(3) $$YBH_{ijk}={\text{θ}}_{0\;}+{\text{θ}}_{1\;}Species_{k}+{\text{θ}}_{2\;}Planting\;year_{i}+{\text{θ}}_{3\;}BGC\;Zone_{i}+{\text{θ}}_{4\;}ASMR_{ij}+u_{i}+v_{ij}+e_{ijk}$$

where *YBH_ijk_* is the years to breast height for tree *k* in plot *j* in site *i*. Model fixed effects beyond the intercept θ_0_ are denoted by: (i) tree species (*Species_k_*); (ii) the year of planting for site *i* (*Planting year_i_*); (iii) the biogeoclimatic zone of the site (*BGC Zone_i_*); and (iv) actual soil moisture regime of the plot (*ASMR_ij_*). The model included two independent Gaussian random effects *u_i_* and *v_ij_* to account for the (pooled) variation among site and plots (nested within site). The residual error is denoted by *e_ijk_.*

##### 2.5.2.3 Radial growth model

Radial growth was quantified from the core samples, calculated as the mean annual basal area increment for each tree over successive 5-year breast height age classes, as follows:


(4) $$BAI_{p}=\;\frac{\sum_{i=1}^{5}BAI_{i}}{5}$$

Where *p* = 5-year period for a given age class, *i*= year 1 to 5 in age class *p*, and BAI_i_ = annual basal area increment during year *i*. Age classes used in this study were as follows: ages 6-10, 11-15, 16-20, and so on.

Similar to the SI and YBH models in this analysis, the BAI model was specific to the study region, and data from all four species were pooled into a single model with the following form:


(5) $$BAI_{ijkl}={\text{θ}}_{0\;}+{\text{θ}}_{1\;}Species_{l}+{\text{θ}}_{2\;}Age\;class_{kl}+{\text{θ}}_{3\;}Prop.Diam._{ijl}+{\text{θ}}_{4\;}BGC\;Zone_{i}+{\text{θ}}_{5\;}ASMR_{ij}+u_{i}+v_{ij}+e_{ijkl}$$

where *BAI_ijkl_* (see Equation 4) is the basal area increment for tree *l* at age class *k* in plot *j* in site *i*. Model fixed effects beyond the intercept θ_0_ are denoted by: (i) tree species (*Species_l_*); (ii) age class (*Age class_kl_*); (iii) the proportion of the tree’s DBH to plot-level quadratic mean diameter at the time of sampling (*Prop.Diam._ijl_*); (iv) the biogeoclimatic zone of the site (*BGC Zone_i_*); and (v) actual soil moisture regime of the plot (*ASMR_ij_*). The *Prop.Diam._ijl_* variable was included as a covariate in the model because radial growth in many tree species is sensitive to competition from neighbouring trees (Fritts, 2012). The random effects structure was same as the YBH model. Since each tree involved multiple basal area increment observations, we modeled autocorrelation among the residuals for each tree with a first-order autocorrelation structure.

## 3 Results

We collected growth data from trees distributed over 173 plots in 25 sites (**Table 1**, **Appendix 2**, **Appendix 3**). Mean stand density was 2075 stems per hectare, ranging from 200 to 5800 (not shown).

**Table 1:** Summary of tree data collected in the study area in central British Columbia.

				Crown class			Tree size			Growth		
Species	Sites	n	Cores	D/C	I	S	Age (years)	Height (m)	DBH (cm)	SI	BAI	YBH
Douglas-fir	7	215	122	145	53	17	21-60	9.2-25.1	12.8-40.5	20.5 (3.1)	885 (505)	11.9 (4.4)
Western larch	17	404	324	349	47	8	13-37	7.2-28.9	7.5-32.2	22.1 (3.5)	766 (422)	5.8 (2.4)
Ponderosa pine	5	133	89	107	25	1	20-33	5.6-16.7	9.8-29.5	20.6 (3.0)	788 (435)	6.0 (2.0)
Lodgepole pine	24	705	440	556	128	21	10-72	6.8-25	5.1-38.9	20.3 (2.7)	800 (403)	7.0 (3.1)

Age, Height and DBH columns report range of individual tree observations across all sites. The SI, BAI and YBH columns report species means, with standard deviations in brackets. DBH, diameter at breast height; n, total number of trees measured; Cores, number of cores collected from trees with a dominant/co-dominant crown class; D/C, number of trees with dominant/co-dominant crown class; I, intermediate crown class; S, suppressed crown class; SI, site index (m); BAI, annual basal area increment (mm²/year); YBH, years for tree to achieve 1.3 m height.

**Figure 1:**
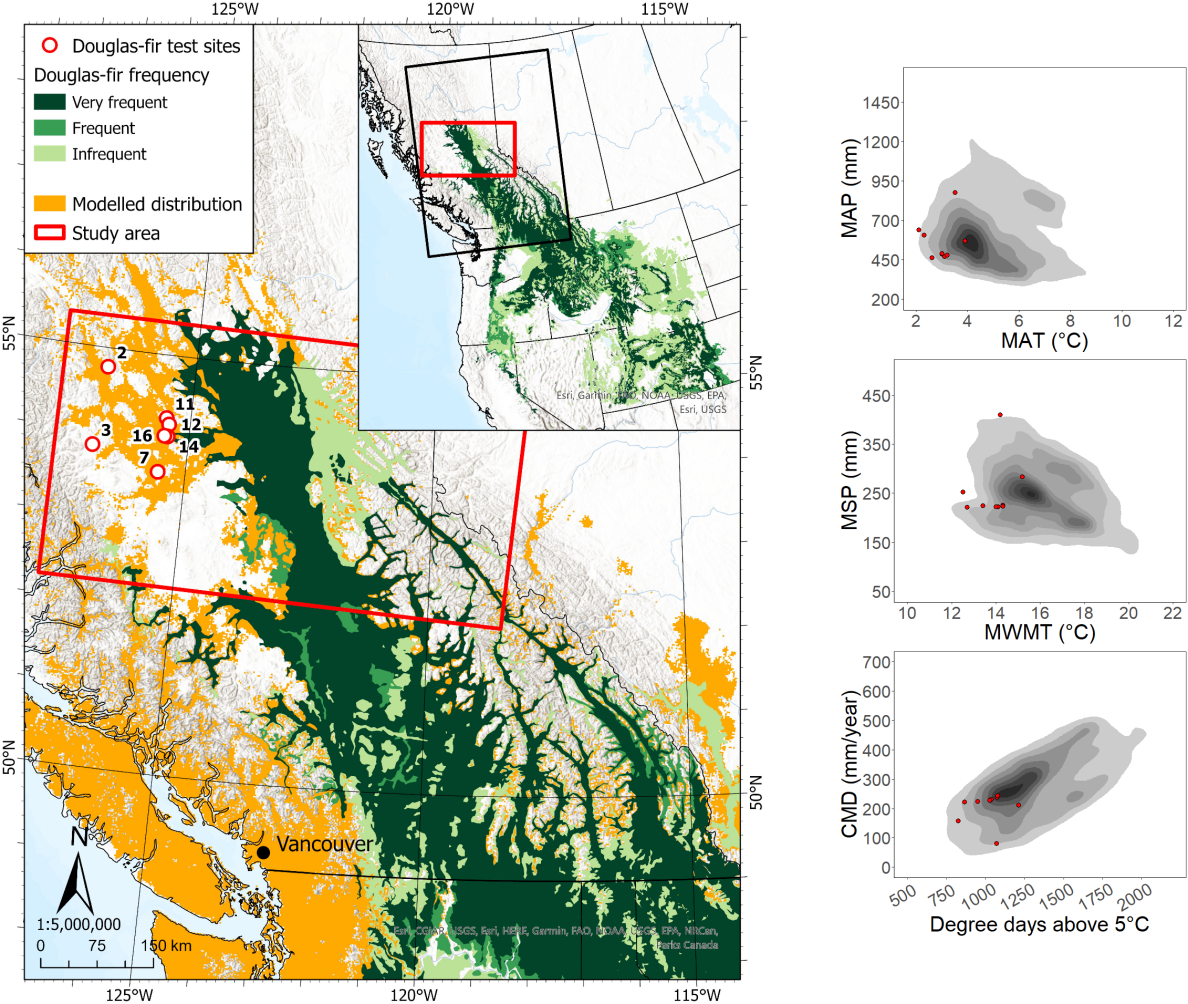
Interior Douglas-fir test site locations and current geographic and climatic ranges. Left panel: test site locations, the species’ current geographic range (CGR) in British Columbia and northwest United States. Test site numbers correspond to site information presented in **Appendix 2**. Species CGR data are from MacKenzie and Mahony (2021); modelled distribution shows the estimated species suitable climatic habitat in 1997-2006 (Gray and Hamann, 2013). Right panels: the 1981–2010 climate normals of the test sites (red dots), along with a kernel density distribution of the species’ climate normals from its distribution in western North America (grey contours). MAP, mean annual precipitation; MAT, mean annual temperature; MSP, mean summer precipitation; MWMT, mean warmest month temperature; CMD, climatic moisture deficit.

The seven sites at which Douglas-fir was planted in the study region were located ca. 20 to 140 km from the nearest edge of the contiguous portion of the species’ current geographic range (**Figure 1**). Six of the sites were located within the modeled species climatic distribution, and the majority were within the species’ actual climate distribution in western North America. In the seven sites, we collected data on a total of 215 Douglas-fir trees, 145 of which had achieved a dominant/co-dominant crown position at the time of sampling (**Table 1**). Ages of dominant/co-dominant trees ranged from 21 to 60, and heights and DBH ranged from 9.2 to 25.1 m and 12.8 to 40.5 cm, respectively.

**Figure 2:**
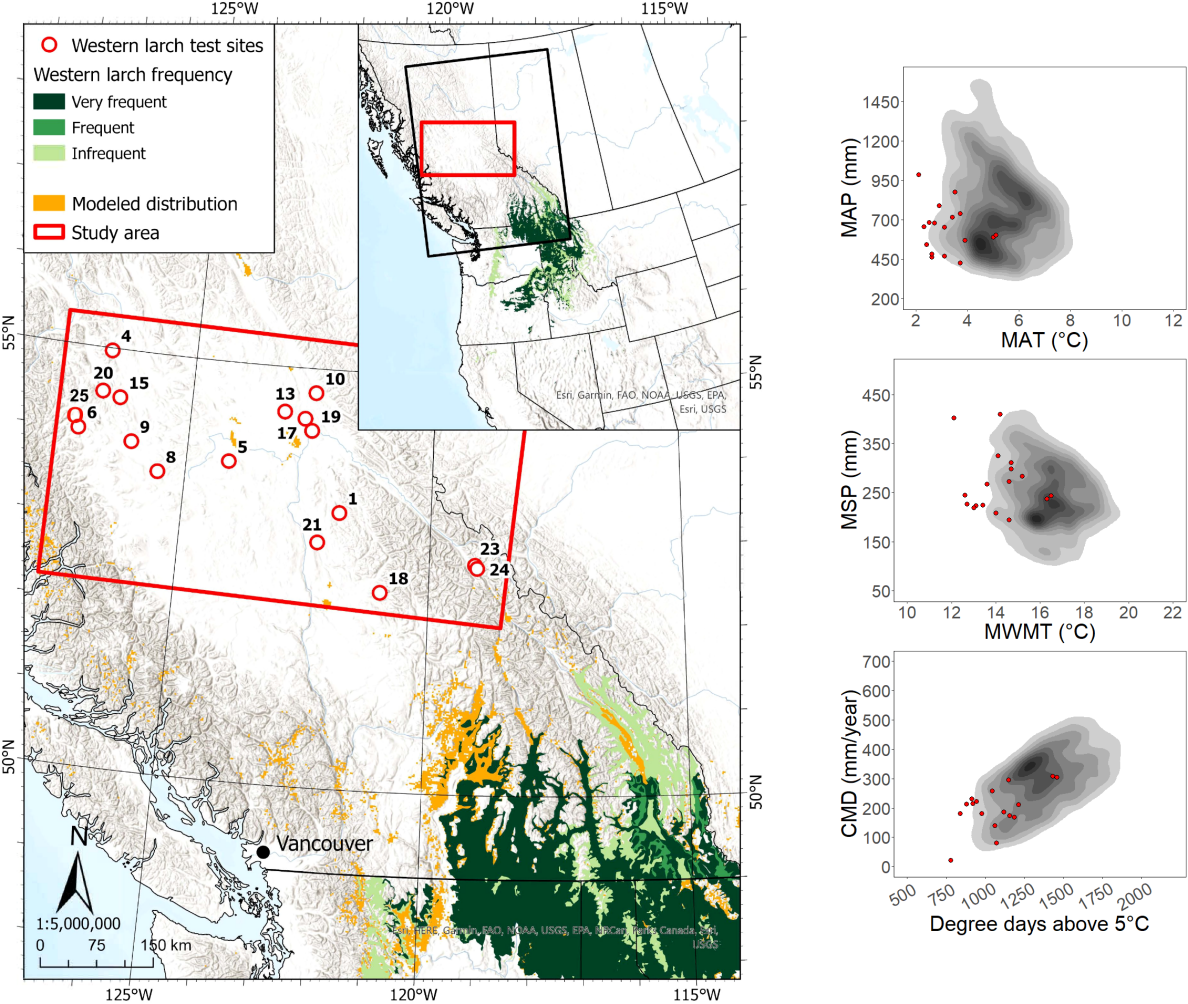
Western larch test site locations and geographic/climatic distributions. See **Figure 1** caption for map details.

The 17 sites planted with western larch in the study area were located ca. 220 to 700 km from the nearest edge of the species’ current geographic range (**Figure 2**). All western larch sites were located outside its modeled species climatic distribution, with approximately half the sites located beyond the cold limit of the species’ actual climate distribution in western North America. Data for 404 western larch trees across the 17 sites showed that most (n = 349) occupied a dominant/co-dominant crown position at the time of sampling, with ages ranging from 13 to 37 years. Heights and DBH of dominant/co-dominant western larch trees in the study area ranged from 7.2 to 28.9 m and 7.5 to 32.2 cm, respectively.

**Figure 3:**
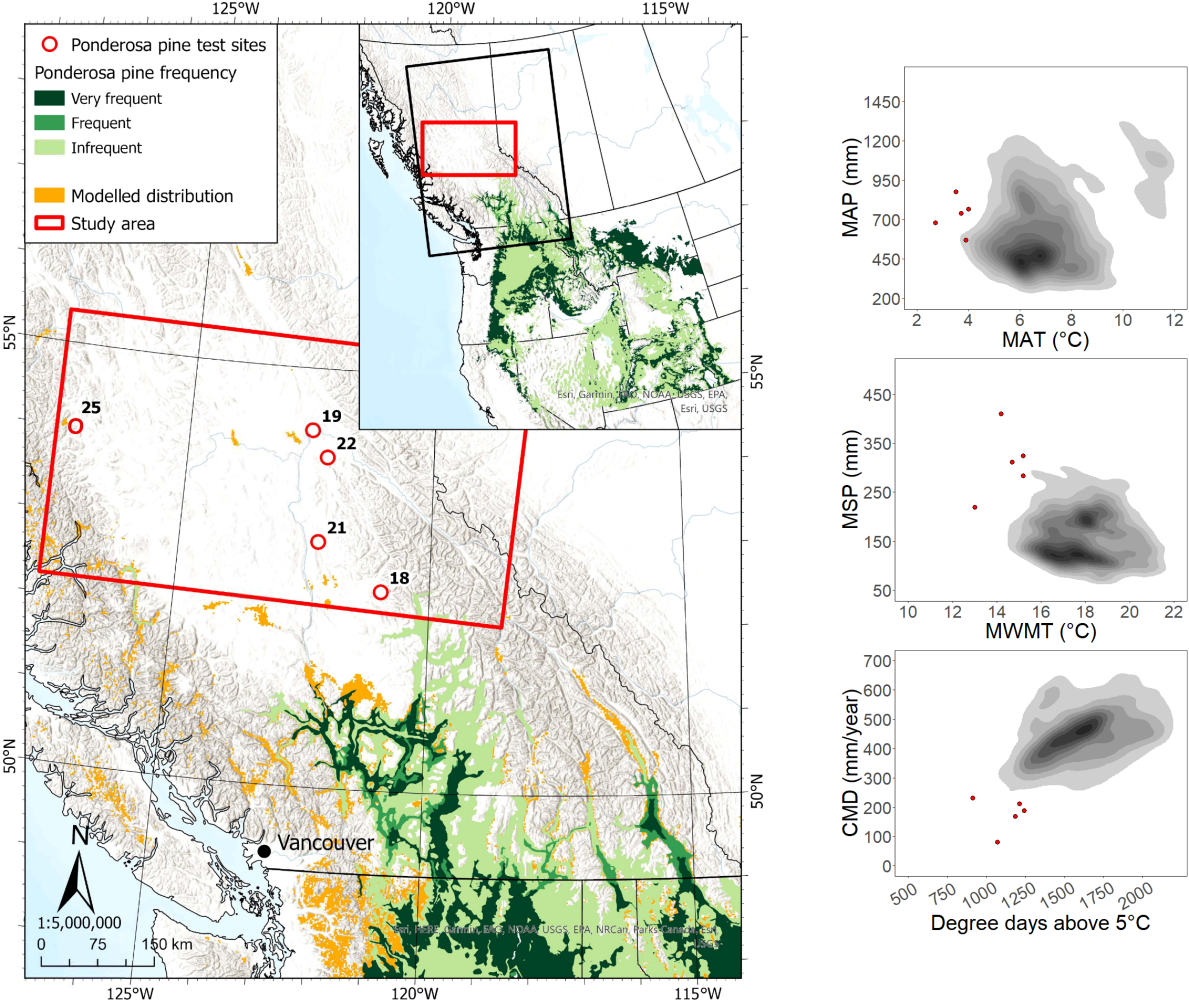
Ponderosa pine test site locations and geographic/climatic distributions. See **Figure 1** caption for map details.

Five sites planted with ponderosa pine in the study region were located ca. 110 to 460 km from the nearest edge of the species’ current geographic range (**Figure 3**). All sites were located outside the modeled species climatic distribution, with approximately half the sites located beyond the cold and wet limit of the species’ actual climate distribution in western North America. Data collected for 133 ponderosa pine trees (107 occupying a dominant/co-dominant crown class) across the study area showed an age range of 20 to 33 years, with heights and DBH ranging from 5.6 to 16.7 m and 9.8 to 29.5 cm, respectively.

### 3.1 Analysis 1 – comparison of productivity inside and outside the species’ current geographic range

**Figure 4:**
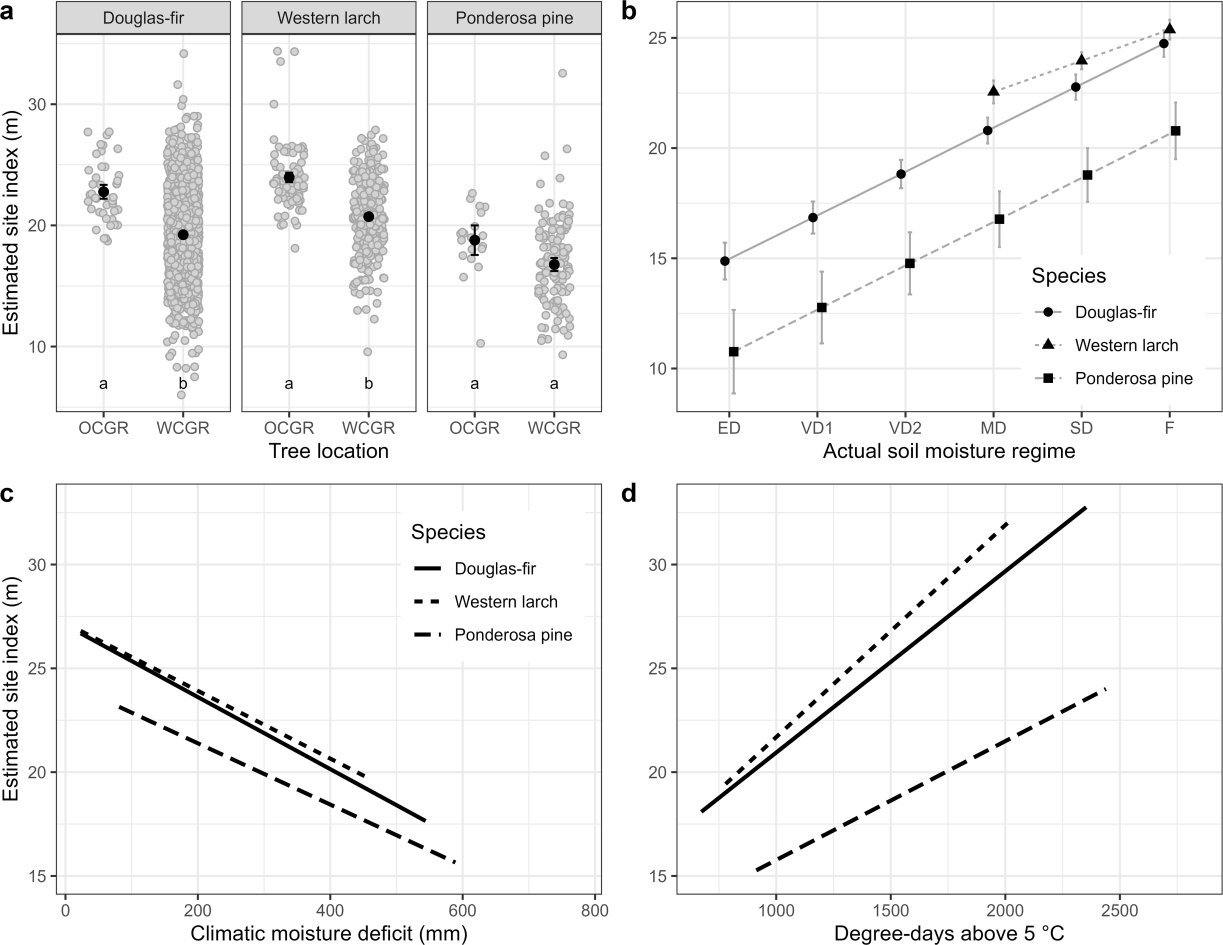
Results from Analysis 1. Estimated site index for Douglas-fir, western larch and ponderosa pine by four effects: **(a)** tree location; **(b)** actual soil moisture regime; **(c)** mean annual climatic moisture deficit and **(d)** mean annual growing degree days above 5°C. Analysis was completed separately for each species. Estimates are from model described in Equation 1 and summarized in **Table 3**. Whiskers in panels **(a, b)** show standard errors. Grey dots in panel **(a)** are partial residuals, jittered to improve legibility. Different lower case letters in panel **(a)** indicate statistically different (i.e., p< 0.05, adjusted using Tukey HSD) group means. OCGR, outside current geographic range; WCGR, within current geographic range. Actual soil moisture regime categories: ED, extremely dry; VD1, very dry 1; VD2, very dry 2; MD, moderately dry; SD, slightly dry; F, fresh. See **Appendix 1** for more information on actual soil moisture regime categories.

Site index of 122 Douglas-fir trees planted OCGR had a mean of 20.5 m, with a standard deviation (SD) of 3.1 m (**Table 1**), compared to a mean site index of 19.7 m (SD = 4.5, n = 1187) for trees located WCGR (**Table 2**). The Douglas-fir site index model (Equation 1) indicated that site index of OCGR trees significantly exceeded that of those growing WCGR by an estimated 3.5 m (with a standard error (SE) of 0.59), controlling for environmental variation between sites (**Table 3**, **Figure 4**). Similar to Douglas-fir, estimated site index for western larch trees planted OCGR (mean = 22.1 m, SD = 3.5, n = 319) exceeded estimates for those growing WCGR (mean = 21.9, SD = 3.8, n = 256) by an estimated 3.2 m (SE = 0.45). In contrast to Douglas-fir and western larch, the site index of ponderosa pine planted OCGR (mean = 20.6 m, SD = 3.0, n = 83) did not differ significantly from the site index of trees planted WCGR (mean = 14.2 m, SD = 4.0, n = 124), controlling for environmental variation. Models also indicated that height growth of all three species increased with site mean growing degree days and decreased with mean drought conditions (as measured by CMD). At the site-level, site index increased with actual soil moisture regime.

**Table 2:** Summary of tree records from the SIBEC database of site index estimates for three study species within their current geographic range in British Columbia.

Species	Number of trees	Age (years)	Height (m)	SI (m)
Douglas-fir	1187	10-145	4.8-42.1	19.7(4.5)
Western larch	256	10-120	7.2-40.7	21.9(3.8)
Ponderosa pine	124	25-227	7.5-38.2	14.2(4.0)

Age and Height columns report ranges across all individual tree records, and SI column reports mean site index (m) with standard deviation in brackets.

**Table 3:** Summary of models comparing site index between populations of species planted outside their current geographic range to populations growing within their current geographic range.

Term	Douglas-fir	Western larch	Ponderosa pine
(Intercept)	6.652 (1.226)***	7.619 (2.028)***	5.583 (2.992)
Fixed effects			
WCGR	-3.541 (0.586)***	-3.243 (0.453)***	-2.011 (1.424)
CMD	-0.017 (0.002)***	-0.016 (0.003)***	-0.015 (0.006)*
DD5	0.009 (0.001)***	0.010 (0.001)***	0.006 (0.002)***
ASMR	1.974 (0.158)***	1.414 (0.294)***	2.004 (0.373)***
Model summary			
DF	4	4	4
DFR	1225	333	138
R²	0.31	0.28	0.39
n	1230	338	143

Analysis was completed separately for each species. Model intercept represents trees growing outside the species’ current geographic range. Model coefficients are reported with standard errors in brackets. Coefficient significance: *** p<0.001; * p<0.05. WCGR, tree’s location is within the species’ current geographic range; CMD, climatic moisture deficit; DD5, growing degree days above 5°C; ASMR, actual soil moisture regime; DF, degrees of freedom; DFR, residual degrees of freedom; R², adjusted variance of proportion explained by model; n, number of observations.

### 3.2 Analysis 2 – comparison of productivity among species in the study area

**Figure 5:**
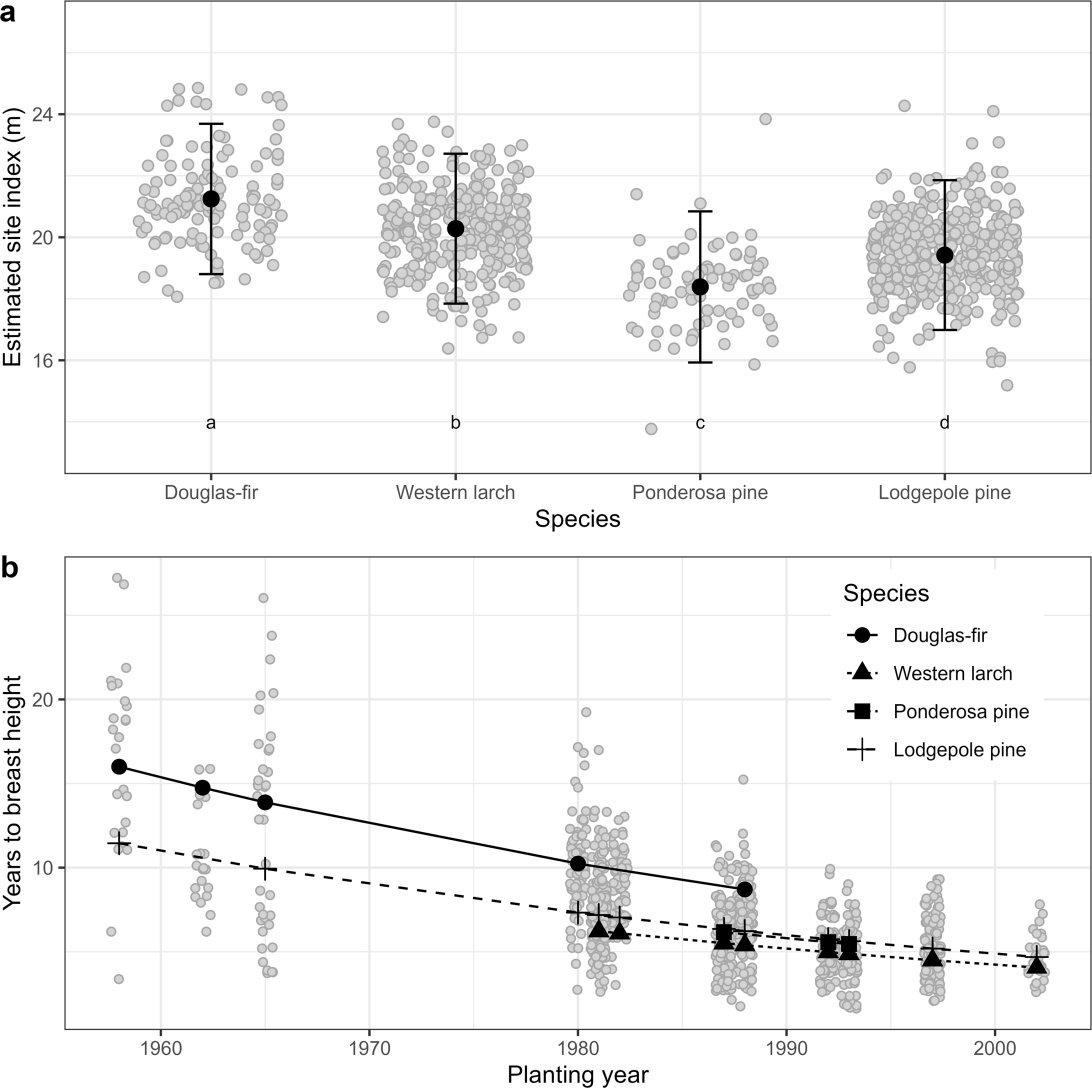
Results from Analysis 2. Panel **(a)** shows estimated site index in the study area by species. Model is described in Equation 2 and summarized in **Table 4**. Whiskers show standard errors. Grey dots are partial residuals, jittered to improve legibility. Different lowercase letters in panel **(a)** indicate statistically different (i.e., p< 0.05, adjusted using Tukey HSD) group means. Panel **(b)** shows estimated years for tree to achieve breast height (i.e., 1.3 m) by species and planting year. Model is described in Equation 3 and summarized in **Table 4**. Grey dots are years to breast height data, jittered to improve legibility.

Analysis of site index of the four tree species in the study area indicated that Douglas-fir site index was higher than the other species and significantly exceeded that of the local species (lodgepole pine) by an estimated 1.8 m (SE = 0.2), controlling for environmental variables (**Figure 5**, **Table 4**, **Appendix 4**). Mean estimates of western larch site index exceeded those of lodgepole pine by an estimated 0.9 m (SE = 0.2). In contrast to Douglas-fir and western larch, site index was significantly lower for ponderosa pine than for lodgepole pine by an estimated 1.0 m (SE = 0.3). Similar to the site index models described in Equation 1, this model indicated that site index for the four species increased with site-level actual soil moisture regime, but within the study region, site index did not vary by site climate.

**Table 4:** Summary of site index, years to breast height, and basal area increment models comparing three species planted outside their current geographic range to a local tree species.

Term	Site index	Years to breast height	Basal area increment
(Intercept)	16.076 (2.564)***	42.141 (7.736)***	4.6670 (0.2573)***
Species			
Douglas-fir	1.826 (0.212)***	0.334 (0.038)***	0.0404 (0.0402)
Western larch	0.859 (0.150)***	-0.143 (0.025)***	-0.3347 (0.0303)***
Ponderosa pine	-1.035 (0.323)**	-0.031 (0.052)	-0.2609 (0.0619)***
Site-level effects			
ICH	3.496 (2.802)	0.155 (0.226)	-0.3592 (0.2470)
SBS	0.411 (2.485)	0.115 (0.201)	-0.3604 (0.2190)
ASMR	1.672 (0.285)***	-0.045 (0.042)	0.2357 (0.0470)***
Tree-level effects			
Age class	–	–	0.0058 (0.0008)***
DBH prop.	–	–	1.4867 (0.0492)***
Planting year	–	-0.020 (0.004)***	–
Variance components			
Component	St.Dev.	St.Dev.	St.Dev.
Site	2.37	0.177	0.190
Site/Plot	1.12	0.171	0.197
Residual	1.47	0.259	0.387
Model summary			
AR (1)	–	–	0.656
n	959	958	4274
R²	0.193	0.408	0.378

Model intercept is for lodgepole pine trees and Engelmann Spruce - Subalpine Fir biogeoclimatic zone. Model coefficients are reported with standard errors in brackets. Coefficient significance: *** p<0.001; ** p<0.01. ASMR, actual soil moisture regime; ICH, Interior Cedar-Hemlock biogeoclimatic zone; SBS, Sub-Boreal Spruce biogeoclimatic zone; DBH prop., proportion of tree’s DBH to plot-level quadratic mean diameter; AR (1), first-order autocorrelation coefficient for residuals (reported for basal area increment model only); St.Dev., standard deviation; n, number of observations in model; R², proportion of variance explained by model fixed effects.

The YBH model (Equation 3) indicated that Douglas-fir planted OCGR had the slowest early growth of the four species in the study area (**Table 4**, **Appendix 4**), taking on average 11.9 years (SD = 4.4) to grow to 1.3 m in height (**Table 1**). The time required to achieve 1.3 m in height increased with stand age for all four species (**Figure 5**); in other words, more recently planted trees had faster early growth than older trees. Results from the BAI model (Equation 5) showed that Douglas-fir radial growth was similar to that of lodgepole pine (**Table 4**, **Appendix 4**). Radial growth for these species exceeded both western larch and ponderosa pine. Similar to the site index model, basal area increment increased significantly with site-level actual soil moisture regime, but did not vary with site climate.

## 4 Discussion

Our finding of high growth rates for all three species outside their current geographic range, outside the current climate distribution for ponderosa pine and partially for western larch, and outside the modelled species’ distribution for western larch and ponderosa pine provides long-term field evidence corroborating models that show the fundamental niche may be far larger than the realized niche (Zhao et al., 2023, 2024; Laughlin and McGill, 2024). Species distribution models based on bioclimate envelope matching often show strong ability to model species suitability within a species’ geographic range or climatic niche (Rehfeldt et al., 2014), however, their predictive abilities can diminish when extrapolating into suitable habitat outside the species’ current range (Charney et al., 2021), or under scenarios of no-analog climates (Veloz et al., 2012), as may be needed when assessing opportunities for assisted range expansion (Zhao et al., 2023, 2024). Further, most species distribution models utilize a binary variable (presence/absence) of species to imply species suitability; our approach of using a continuous variable complements typical species distribution models by analyzing variation in species growth across ecological gradients (Zhao et al., 2024). The height and radial growth metrics used in this study provide an index of tree fitness (Oliver and Larson, 1996; Fritts, 2012), and site index is a conventional method to estimate the potential growth of a tree species (Oliver and Larson, 1996). Together, these findings suggest that suitable environments for a species may be underestimated by the current geographic range, the current climate distribution, and by species distribution models developed with presence/absence data. These results not only lend support for assisted range expansion; they provide a quantitative measure of suitability closely related to fitness that can be used to facilitate species selection decisions.

Data for all three species suggest that they are capable of similar height growth in environments outside their realized niche, compared to growth of these species within their current geographic range. While this suggests that locations of highest productivity for these species may occur in areas outside, and colder than, their realized niche, we recommend these results be interpreted with caution for a number of reasons. First, the climates of almost all the Douglas-fir sites and approximately half of the western larch sites were located inside the current climate niche that these species occupy in western North America, despite the sites being located outside the species’ current geographic range. Secondly, site index estimates are based on height-age models developed for trees located within the species’ current geographic range, and it is possible that the equations used in this study do not adequately describe height-age relationships of trees planted outside these environments. Further, we only analyzed data from British Columbia, which encompasses the northern distributions for these species; a broader examination of productivity between realized and fundamental niches would be obtained by including data from the full species’ ranges in western North America. Nevertheless, our results do imply that all three species are capable of at least similar growth rates outside, and north of, their realized niche, and that the limits of their range (i.e., their fundamental niche) are yet to be identified (Booth, 2024; Laughlin and McGill, 2024). Douglas-fir has an especially wide ecological amplitude (Klinka et al., 1999) and has been successfully established across a range of climates around the world (Hermann and Lavender, 1999). While all three species are commonly found in semi-arid environments in BC (Klinka et al., 1999), it appears that their highest growth rates may occur in sites where soil moisture is not limited, even if the climate is colder than their realized niche. Some of the trials were established during the cold Pacific Decadal Oscillation phase of 1945–1977 when winter temperatures in the study area were significantly colder than during the 1981–2010 climate normal period used in this study (Moore, 1991; Macias Fauria and Johnson, 2008). Thus, these species may have the adaptive capacity to establish and maintain productivity in climates currently colder than those of our study region.

Our results also demonstrate that across all sites in the study area, the growth of Douglas-fir and western larch historically exceeded that of lodgepole pine, a species naturally found in the study area and extensively used for reforestation due to fast juvenile growth. Douglas-fir and western larch growth may increasingly exceed that of lodgepole pine in the future if climate change exceeds the tolerances of lodgepole pine, ignoring possible mitigation to lodgepole pine site index decreases through assisted population migration (Wang et al., 2006). While the growth of ponderosa pine was slower than lodgepole pine, we note that most of the ponderosa pine trees evaluated in this study had achieved a dominant/co-dominant crown class, suggesting they are capable of competitive growth rates, especially if planted in pure stands. The three species are considered well-adapted to drought, and their current ranges include regions of western North America that typically experience hotter temperatures and drier conditions than the study region. Thus, they may be suitable species for reforestation on drought-prone sites in the study region or where local species are considered at high risk of drought stress or mortality (DeLong et al., 2019; MacKenzie and Mahony, 2021).

By considering two additional measures of productivity, (i.e., the time for a tree seedling to achieve breast height, and radial growth increment), we were able to identify further important aspects related to species suitability. Douglas-fir had the highest site index of all species in this study; however, these trees took significantly longer to achieve 1.3 m in height than the other three species in the study area. Slow initial growth of Douglas-fir has been observed near its northern range limits (DeLong, 1996; Jull, 1996) and may be related to a high sensitivity to frost and cold soils during initial establishment. This also means that where Douglas-fir is planted in intimate mixtures with species that have faster initial growth, it may become overtopped and suppressed if shading sufficiently suppresses growth. While Douglas-fir is considered to have moderate shade tolerance (Klinka et al., 1999), there are occurrences of plantations in this region where lodgepole pine has outcompeted Douglas-fir within the first few years of planting, resulting in suppression and growth losses (unpublished data). Thus, while long-term Douglas-fir productivity may make it a desirable timber species in the future, forest managers need to be aware that species such as Douglas-fir planted outside its range may require additional measures such as shelterwood silviculture systems, or competition control to establish them successfully on certain sites. Conversely, shade-intolerant tree species such as western larch and ponderosa pine should be planted promptly after harvest, before vegetation competition has become established (Klinka et al., 1999). Controlling for year of planting and species, we did not detect any climatic or site factors controlling initial tree growth. However, factors such as frost and vegetation competition can delay or prevent trees from achieving target heights, and these are generally higher on lower slope positions, co-occurring with higher soil moisture availability and richer nutrient regimes (Steen et al., 1990). Within the study area, the early growth of all species has accelerated over time. This may be related to a combination of factors including improvements in the tree seedlings themselves; improved reforestation practices, or climate change over the past decades that has improved growing conditions.

The strong link between growing degree days and tree site index found in this study has also been established for other species and other regions (Nigh, 2006). This variable is strongly correlated with mean annual temperatures and winter temperatures in our study sites (r > 0.9, not shown), and suggests that continued temperature increases (and thus increases in growing degree days) in the study region may result in increased potential height growth of the three species studied here. However, our results also show that sites with lower soil moisture were associated with slower height growth for all three species. Actual soil moisture regime was a strong predictor of height and radial growth for all species studied, and across locations both outside and within the current geographic range. Strong positive relationships between soil moisture regime and site index have been described in this area for white spruce (Wang and Klinka, 1996), and our results suggest a similar relationship exists for the species studied here, even where planted in regions colder than their current range. Thus, the long-term trajectories for height growth in all species remains unclear due to the potential confounding effects of increasing growing degree days and possible decreasing soil moisture related to higher evapotranspiration and severe drought events (DeLong et al., 2019; Zhao et al., 2020).

While our data allowed us to quantify the growth potential of the studied tree species, our study does not quantify tree survival, as data required for an analysis of survival were only available for three sites. Thus, our results can be used only to infer the growth potential of non-local tree species once they are successfully established. The challenge for assisted range expansion of tree species is to identify sites where they can become established now and are predicted to be well suited to projected future climates. A site-specific climate change informed species selection tool already exists for British Columbia that provides modelled guidance (MacKenzie and Mahony, 2021, https://bcgov-ffec.ca/cciss/). However, from a silvicultural perspective, the ability to successfully establish non-local species in the current period is an acute information need. Our data demonstrate that successful establishment is possible on some sites, but cannot suggest the breadth of suitable conditions or other constraints. Knowledge of the ecology and silvics of candidate species for assisted range expansion is required to guide decisions as to their deployment in novel areas. Further work to quantify tree survival across a range of sites outside the species’ distribution, as well as to understand the role of non-climatic factors such as ectomycorrhizal fungal associations on tree vigour are important areas for further research. Potential ecological risks associated with assisted range expansion of tree species may be somewhat mitigated when the introduced species originate from ecologically and geographically adjacent locations and are often functionally equivalent to the local species (Buckley and Catford, 2016).

Tree seed source can strongly influence productivity of planted stands (Leites et al., 2012; Sattler et al., 2024) and accounting for provenance climate would yield additional insights into performance when planted outside their current range (Wang et al., 2006; O’Neill et al., 2008). We did not include provenance in our analyses because seed source data were not available for all sites, and preliminary analysis indicated the climatic transfer distance was generally insufficient to yield robust relationships between provenance and site climate. Nevertheless, our results demonstrate the utility of small, informal tree species trials, even where they lack seed source data, to generate data that can be used to quantify tree fitness across geographic and ecological gradients. We support the recommendation of others to utilize existing field trials to enhance our understanding of tree fundamental niches (LePage and McCulloch, 2011; Booth, 2024). Notwithstanding the need to ensure ecological suitability of species moved outside their current geographic range, methods illustrated here overcome some of the limitations of bioclimate niche-based species distribution models when considering assisted range expansion. By providing a quantifiable metric with which to evaluate species suitability, practitioners and researchers in fields of conservation, restoration, forestry, agriculture, and horticulture will be better able to assess alternative species options for future climates.

## 5 Conclusions

This study demonstrates that observations from multiple, often informal tree species trials, when analyzed collectively, can improve our understanding of a species’ fundamental niche and its amenability to assisted range expansion. All three species in this study demonstrated high productivity when planted outside their current geographic range, suggesting that their fundamental niche has historically included climates colder than their realized niche. Further, these species demonstrated growth rates that exceed or are similar to those of a conventional tree species in the study region, suggesting that in certain environments they can remain competitive with locally adapted species. In areas that are projected to experience increases in drought frequency or magnitude, species such as those studied here may be viable reforestation options, even when the current climate is colder than their realized niche. The productivity of these species varied consistently across ecological gradients and soil moisture regime was a consistent and strong predictor of growth in all three species. This suggests that under climate change, the productivity of these conifers in British Columbia may be primarily influenced by changes in precipitation and available soil moisture.

## Data Availability

The raw data supporting the conclusions of this article will be made available by the authors, without undue reservation.
