# Supplementary material for: High productivity of tree species planted outside their current geographic range indicates large regions of unrealized niche space
Source: Front Plant Sci. 2025 Aug 28;16:1650428. doi: 10.3389/fpls.2025.1650428 (PMC12424236; doi:10.3389/fpls.2025.1650428)
Supplement: Supplementary file 1 [file Table1.docx]

Supplementary Material

Appendix 1: Actual soil moisture regime categories used in this study. Abbreviations: AET:PET = mean annual ratio of actual evapotranspiration to potential evapotranspiration.

| Class | AET:PET | Soil moisture deficit |
| --- | --- | --- |
| Excessively dry (ED) | < 0.55 | Deficit > 5 months |
| Very dry 1 (VD1) | ≥ 0.55 < 0.65 | Deficit > 4 months and ≤ 5 months |
| Very dry 2 (VD2) | ≥ 0.65 < 0.75 | Deficit > 3 months and ≤ 4 months |
| Moderately dry (MD) | ≥ 0.75 < 0.85 | Deficit > 2 months and ≤ 3 months |
| Slightly dry (SD) | ≥ 0.85 < 0.95 | Deficit > 1.5 months and ≤ 2 months |
| Fresh (F) | ≥ 0.95 < 1.0 | Deficit rarely occurs. |
