# Supplementary material for: High productivity of tree species planted outside their current geographic range indicates large regions of unrealized niche space
Source: Front Plant Sci. 2025 Aug 28;16:1650428. doi: 10.3389/fpls.2025.1650428 (PMC12424236; doi:10.3389/fpls.2025.1650428)
Supplement: Supplementary file 2 [file Table2.docx]

Supplementary Material

Appendix 2: Summary of study sites. Abbreviations: Lat. = latitude (°N); Long. = longitude (°W); Elevation = elevation above sea level (m); BGC = biogeoclimatic variant (Meidinger and Pojar 1991); Plots = number of 50m² plots established in site.

| Site | Lat. | Long. | Elevation | BGC | Plots | Planting year |
| --- | --- | --- | --- | --- | --- | --- |
| 1 | 54.4650 | 123.0863 | 833 | SBSmk1 | 4 | 1997 |
| 2 | 54.2604 | 122.5020 | 737 | SBSmk1 | 7 | 1993 |
| 3 | 53.9474 | 122.1707 | 715 | SBSwk1 | 4 | 1993 |
| 4 | 54.3978 | 122.6589 | 764 | SBSmk1 | 7 | 1997 |
| 5 | 54.7113 | 122.4698 | 798 | SBSwk1 | 6 | 1997 |
| 6 | 52.9367 | 122.2442 | 898 | SBSmw | 11 | 1987 |
| 7 | 54.7718 | 126.8239 | 1,064 | SBSmc2 | 9 | 1958 |
| 8 | 54.9708 | 126.7672 | 952 | SBSmc2 | 5 | 1982 |
| 9 | 54.0413 | 125.5038 | 824 | SBSdk | 6 | 1988 |
| 10 | 54.0368 | 125.4348 | 848 | SBSdk | 6 | 1988 |
| 11 | 53.8171 | 124.1396 | 1,054 | SBSmc2 | 9 | 1982 |
| 12 | 54.1788 | 125.4375 | 876 | SBSdk | 6 | 1962 |
| 13 | 54.1796 | 125.4341 | 896 | SBSdk | 12 | 1965 |
| 14 | 53.6046 | 125.5522 | 1,033 | SBSdk | 6 | 1980 |
| 15 | 53.6045 | 125.5523 | 1,039 | SBSdk | 6 | 1981 |
| 16 | 53.9266 | 126.1480 | 1,022 | SBSmc2 | 5 | 1992 |
| 17 | 53.8335 | 126.9297 | 1,015 | SBSmc2 | 6 | 1980 |
| 18 | 54.4283 | 126.4846 | 811 | SBSdk | 5 | 1981 |
| 19 | 54.0158 | 127.2566 | 1,003 | SBSmc2 | 5 | 1981 |
| 20 | 54.4803 | 126.8532 | 645 | SBSdk | 5 | 1981 |
| 21 | 53.3038 | 121.8421 | 1,339 | ESSFwk1 | 7 | 1988 |
| 22 | 52.3807 | 120.9552 | 1,116 | ICHwk2 | 13 | 1987 |
| 23 | 52.7456 | 119.0963 | 808 | ICHmm1 | 8 | 2002 |
| 24 | 52.7088 | 119.0527 | 781 | ICHmm1 | 6 | 1981 |
| 25 | 54.0208 | 127.4025 | 945 | SBSmc2 | 9 | 1992 |
