# Supplementary material for: High productivity of tree species planted outside their current geographic range indicates large regions of unrealized niche space
Source: Front Plant Sci. 2025 Aug 28;16:1650428. doi: 10.3389/fpls.2025.1650428 (PMC12424236; doi:10.3389/fpls.2025.1650428)
Supplement: Supplementary file 3 [file Table3.docx]

Supplementary Material

Appendix 3: Summary of tree species planted at study sites. Columns DBH, Height, Age and SI report mean with standard deviation in brackets. Abbreviations: PSME = Douglas-fir; LAOC = western larch; PIPO = ponderosa pine; PICO = lodgepole pine.

| Site | Species | n | DBH (cm) | Height (m) | Age (years) | SI (m) |
| --- | --- | --- | --- | --- | --- | --- |
| 1 | LAOC | 22 | 12.9(2.6) | 11.7(1.1) | 21.8(0.8) | 22.0(1.3) |
|  | PICO | 28 | 10.5(2.5) | 10.7(0.8) | 18.4(0.9) | 21.8(1.0) |
| 2 | LAOC | 20 | 18.6(3.8) | 14.2(1.5) | 25.2(2.0) | 23.5(1.8) |
|  | PICO | 23 | 15.0(2.5) | 12.5(1.0) | 23.5(2.0) | 20.9(1.2) |
|  | PIPO | 26 | 17.1(3.3) | 11.7(1.7) | 24.6(1.2) | 20.9(2.4) |
| 3 | PICO | 27 | 14.2(1.9) | 13.5(1.1) | 23.1(0.7) | 22.5(1.4) |
|  | PIPO | 12 | 13.0(2.1) | 11.2(0.6) | 23.2(1.5) | 20.8(1.0) |
| 4 | LAOC | 19 | 14.1(3.4) | 12.4(1.7) | 21.1(0.9) | 23.4(2.3) |
|  | PICO | 25 | 16.0(2.6) | 11.8(1.0) | 19.6(1.9) | 22.4(1.5) |
| 5 | LAOC | 20 | 12.0(3.3) | 11.3(2.6) | 20.8(1.1) | 21.9(3.4) |
|  | PICO | 36 | 10.8(3.0) | 10.1(0.7) | 18.0(1.8) | 21.0(1.2) |
| 6 | LAOC | 20 | 17.3(3.2) | 15.2(1.4) | 30.5(1.1) | 21.9(1.4) |
|  | PICO | 22 | 17.9(2.8) | 16.6(1.3) | 31.0(2.1) | 22.3(1.3) |
|  | PIPO | 21 | 19.4(3.6) | 14.0(1.0) | 30.2(1.3) | 21.1(1.0) |
| 7 | PICO | 18 | 22.8(7.4) | 17.6(3.6) | 51.4(10.6) | 18.3(1.5) |
|  | PSME | 25 | 24.9(6.2) | 19.2(2.5) | 46.4(6.0) | 20.0(2.5) |
| 8 | LAOC | 20 | 23.4(3.9) | 16.2(1.3) | 31.8(0.7) | 22.4(1.5) |
|  | PICO | 21 | 19.6(2.7) | 14.8(1.3) | 29.6(0.9) | 21.0(1.5) |
| 9 | PICO | 21 | 18.2(3.5) | 14.9(1.2) | 25.6(1.5) | 23.1(1.4) |
|  | PSME | 20 | 18.2(2.5) | 14.0(1.1) | 26.0(0.6) | 23.2(1.6) |
| 10 | PICO | 20 | 17.6(3.7) | 13.5(1.3) | 26.4(2.9) | 21.0(1.3) |
|  | PSME | 20 | 18.0(2.3) | 12.2(1.2) | 23.8(1.3) | 21.9(1.8) |
| 11 | LAOC | 20 | 21.2(4.2) | 12.7(1.6) | 29.7(1.9) | 19.1(1.8) |
|  | PICO | 23 | 17.9(3.4) | 12.2(1.5) | 31.9(2.5) | 16.9(1.6) |
| 12 | PICO | 16 | 18.0(3.4) | 17.1(1.5) | 58.0(1.2) | 15.0(1.3) |
|  | PSME | 20 | 22.3(4.5) | 16.7(1.5) | 48.4(2.8) | 17.1(1.7) |
| 13 | PICO | 23 | 20.7(4.6) | 15.5(2.0) | 49.6(8.6) | 15.9(2.3) |
|  | PSME | 19 | 20.4(4.6) | 15.1(1.6) | 39.3(3.0) | 18.1(2.0) |
| 14 | PICO | 21 | 15.2(2.9) | 13.4(2.0) | 31.6(1.0) | 18.6(2.6) |
|  | PSME | 21 | 18.6(4.1) | 12.5(2.0) | 27.9(2.6) | 19.8(2.3) |
| 15 | LAOC | 13 | 14.3(4.1) | 12.1(1.9) | 28.8(1.3) | 18.7(1.3) |
|  | PICO | 22 | 14.5(3.1) | 12.9(2.3) | 31.1(2.1) | 17.9(2.6) |
| 16 | LAOC | 20 | 15.9(3.7) | 10.2(1.7) | 23.4(1.2) | 18.9(2.0) |
|  | PICO | 20 | 13.5(3.2) | 9.6(1.3) | 22.2(1.6) | 17.7(1.5) |
| 17 | PICO | 20 | 19.6(2.9) | 15.6(1.1) | 33.8(2.2) | 20.2(1.3) |
|  | PSME | 20 | 25.4(4.2) | 16.7(1.3) | 30.3(1.4) | 24.4(1.9) |
| 18 | LAOC | 21 | 19.6(4.4) | 16.7(1.2) | 32.6(1.5) | 22.7(1.3) |
|  | PICO | 28 | 15.6(3.7) | 16.6(1.1) | 31.6(2.5) | 22.4(1.4) |
| 19 | LAOC | 22 | 17.8(3.7) | 13.4(1.5) | 33.0(1.2) | 18.8(1.7) |
|  | PICO | 23 | 18.2(3.9) | 13.1(1.4) | 33.2(1.7) | 17.5(1.6) |
| 20 | LAOC | 20 | 24.6(4.8) | 24.2(2.3) | 35.0(1.5) | 29.9(2.1) |
| 21 | LAOC | 24 | 20.0(3.2) | 14.4(1.2) | 26.3(1.0) | 22.9(1.5) |
|  | PICO | 25 | 19.3(3.3) | 14.4(0.8) | 30.2(1.0) | 20.3(1.0) |
| 22 | LAOC | 22 | 18.2(3.7) | 15.3(1.2) | 27.8(0.9) | 23.5(1.3) |
|  | PICO | 22 | 15.3(2.6) | 14.9(1.0) | 26.6(0.8) | 22.7(1.2) |
|  | PIPO | 26 | 21.6(4.3) | 14.3(1.1) | 25.7(1.6) | 23.2(1.7) |
| 23 | LAOC | 22 | 12.8(2.6) | 10.3(1.8) | 14.1(0.7) | 25.8(2.2) |
|  | PICO | 23 | 10.0(2.7) | 8.3(1.2) | 12.5(1.3) | 22.6(1.7) |
| 24 | LAOC | 20 | 20.0(3.4) | 17.1(1.8) | 32.6(1.2) | 23.1(1.9) |
|  | PICO | 21 | 17.4(3.5) | 17.6(1.2) | 33.4(1.5) | 22.8(1.1) |
| 25 | LAOC | 24 | 17.8(2.7) | 10.9(1.4) | 28.7(1.3) | 17.4(1.9) |
|  | PICO | 22 | 18.2(3.3) | 12.7(1.9) | 28.5(1.5) | 19.7(1.1) |
|  | PIPO | 22 | 18.0(2.9) | 9.4(1.5) | 27.7(0.8) | 15.9(1.8) |
