# Supplementary material for: High productivity of tree species planted outside their current geographic range indicates large regions of unrealized niche space
Source: Front Plant Sci. 2025 Aug 28;16:1650428. doi: 10.3389/fpls.2025.1650428 (PMC12424236; doi:10.3389/fpls.2025.1650428)
Supplement: Supplementary file 4 [file Table4.docx]

Supplementary Material

Appendix 4: Summary of model contrasts from Analysis 2. Estimated contrasts are from models summarized in Table 4, with standard error in brackets. Coefficient significance: *** p<0.001; ** p<0.01 (all p values adjusted using Tukey HSD). Abbreviations: SI = site index (m); BAI = basal area increment (mm²/year); YBH = years to achieve breast-height; PSME = Douglas-fir; LAOC = western larch; PIPO = ponderosa pine; PICO = lodgepole pine.

| Effect | Contrast | SI | YBH | BAI |
| --- | --- | --- | --- | --- |
| Species | PICO-PSME | -1.83(0.21)*** | -0.33(0.04)*** | 0.96(0.04) |
|  | PICO-LAOC | -0.86(0.15)*** | 0.14(0.03)*** | 1.40(0.04)*** |
|  | PICO-PIPO | 1.03(0.32)** | 0.03(0.05) | 1.30(0.08)*** |
|  | PSME-LAOC | 0.97(0.26)** | 0.48(0.05)*** | 1.46(0.07)*** |
|  | PSME-PIPO | 2.86(0.39)*** | 0.37(0.06)*** | 1.35(0.10)*** |
|  | LAOC-PIPO | 1.89(0.33)*** | -0.11(0.05) | 0.93(0.06) |
